# Biomarker Discovery for Autism Prediction Using Massive Feature Extraction Based on EEG Signals

**DOI:** 10.3390/s26061862

**Published:** 2026-03-16

**Authors:** Nauman Hafeez, Abdul Rehman Aslam, Muhammad Awais Bin Altaf

**Affiliations:** 1 Center for Neuroimaging Sciences, Institute of Psychiatry, Psychology and Neuroscience, King’s College London, London SE5 8AA, UK; 2 Department of Computer Engineering, University of Engineering and Technology, Taxila 47050, Pakistan; arehman.aslam@uettaxila.edu.pk; 3 Department of Electrical and Computer Engineering, Western Washington University, Bellingham, WA 98225, USA; altafm@wwu.edu

**Keywords:** autism, electroencephalograph (EEG), massive feature extraction, highly comparative time-series analysis (HCTSA), hybrid feature selection, machine learning, explainability, Shapley Additive Explanations (SHAP)

## Abstract

Autism spectrum disorder (ASD) is a heterogeneous neurodevelopmental disorder that requires early diagnosis for better intervention. However, current clinical behavioural examinations are time-consuming and prone to human error. Objective and effective biomarkers are essential for the diagnosis and prognosis of the disorder. Electroencephalography (EEG) is a non-invasive and inexpensive brain-imaging technique that is widely applied in the diagnosis of ASD. Feature-based methods have shown better performance in EEG-based applications. Here, we present a prediction framework based on massive feature extraction using the highly comparative time-series analysis (HCTSA) method and a hybrid feature selection method for the classification of ASD from resting-state EEG. Machine-learning models are trained and tested on a different number of selected features. Our models demonstrated 100% accuracy with ≥50 features on a balanced dataset of 56 participants. The most discriminating EEG channels and features were used in the prediction process, as well as those using Shapley values to provide explainability of our framework. Whilst these results are promising, we acknowledge the limitations of a single small-scale dataset and emphasise the need for validation on larger independent cohorts before clinical translation.

## 1. Introduction

Autism spectrum disorder (ASD) is a neurodevelopmental disorder characterised by social and cognitive impairments, repetitive behaviour, restrictive interests, communication and language problems, stereotypical movements, and sensory problems [1]. It has a complex aetiology, and symptoms vary widely. ASD affects roughly 1–2% of the population, and its prevalence has increased in the last few decades in developed countries. The lower figures reported in developing or least developed countries are generally attributed to underdiagnosis, suggesting that the true biological burden is substantial worldwide [2]. Its complete cure is not yet possible; however, early diagnosis and intervention can help reduce its symptoms and improve quality of life. The Autism Diagnostic Observation Schedule—2nd Edition (ADOS-2) uses cognitive scores for ASD diagnosis. It is considered a gold standard diagnostic mechanism in clinics. However, it is not capable of making a timely diagnosis [3].

Neuroimaging techniques have revealed several structural and functional abnormalities of the brain in ASD patients. Magnetic resonance imaging (MRI) studies have documented differences in cortical thickness, surface area, and white matter integrity [4]. Functional MRI studies have identified altered connectivity patterns, especially in networks dealing with social cognition and language processing [5]. However, MRI scans are expensive, unaffordable, and not readily accessible in many clinical settings, especially in middle- and low-income countries.

Electroencephalography (EEG) is a non-invasive, non-radioactive, and inexpensive brain-imaging technique being extensively researched for cognitive neurological disorder (CND) diagnosis [6]. It has a huge potential for low cost and portability for CND diagnosis [7]. EEG signals provide more information than traditional approaches for ASD prediction using machine learning and deep learning. An automated EEG-based diagnostic tool is required to aid clinicians and improve efficiency, which is based on stable and effective biological markers. A growing body of research has explored EEG-based biomarkers for ASD, focusing on various signal properties [8], including resting-state power spectra [9], event-related potentials [10], and connectivity measures [11].

Several studies have contributed to the goal of ASD detection using resting-state EEG. For example, ref. [12] presented an automated framework for ASD detection using deep-learning models and different time–frequency distribution spectrograms, achieving 97.35% classification accuracy from a 16-subject dataset. Another study used a hybrid graph convolutional network for ASD diagnosis from resting-state EEG signals, attaining an accuracy of 85.12% [13]. Instead of using deep learning, Rogala et al. [14] utilised handcrafted features from the frequency and brain connectivity domain and analysed machine-learning model performance on different metrics, achieving a maximum accuracy of 86% on a balanced 36-subject dataset. However, unlike the first two studies, they have also included an explanation of their models.

Recently, Al-Qazzaz et al. [15] presented multidisciplinary apprach evaluating ASD severity using both statistical methods (ANOVA, Pearson correlation) and artificial-intelligence frameworks (decision trees and LSTM networks), achieving 73.3% accuracy in classifying mild, moderate and severe ASD from typically developing children. This work demonstrates the value of combining traditional statistical analysis with deep learning for EEG-based ASD assessment. Additionally, advances in efficient AI architectures have shown promise for real-time BCI applications. Wu et al. [16] explored the integration of computationally efficient large language models with brain–computer interfaces, highlighting the potential for improved signal processing and reduced computational requirements in EEG-based applications.

Mentioned studies and other similar studies [17,18,19] have limitations such as small sample sizes, leading to poor generalisation, the lack of explainability in deep-learning models, and the need for higher validated classification accuracy using a multi-domain feature set for clinical relevance.

Recent work has also explored dynamic functional connectivity (dFC) approaches, such as the principal subspace of dFC [20], which has shown strong discriminative power in ASD and could enhance future versions of prediction models. Additionally, EEG effective connectivity approaches provide deeper insights into causal interactions within brain-disorder-related networks [21,22] and may complement feature-based prediction frameworks. Whilst these connectivity-based methods show promise, the current study focuses on a comprehensive multi-domain feature-extraction approach to establish a baseline prediction framework with high explainability.

We have addressed several gaps and proposed a comprehensive EEG-based ASD detection pipeline using 56 subjects in this paper. We have also incorporated a massive feature-extraction engine and a hybrid feature-selection approach. Our approach differs from existing methods in several key aspects. First, while most previous studies extract features from one or two specific domains (e.g., frequency-domain power spectra or connectivity metrics), our HCTSA-based approach systematically evaluates over 7000 features per channel, spanning more than 10 domains, avoiding the potential bias of hypothesis-driven feature selection. This comprehensive approach allows the data-driven discovery of unexpected biomarkers that might be missed by pre-selecting specific feature types. Second, our hybrid feature-selection method combines three complementary strategies (variance filtering, statistical testing, and machine-learning-based recursive elimination), providing more robust feature identification than single-method approaches. Third, unlike many black-box deep-learning models, our framework explicitly prioritises interpretability through SHAP analysis, making it more suitable for clinical acceptance.

**Figure 1:**
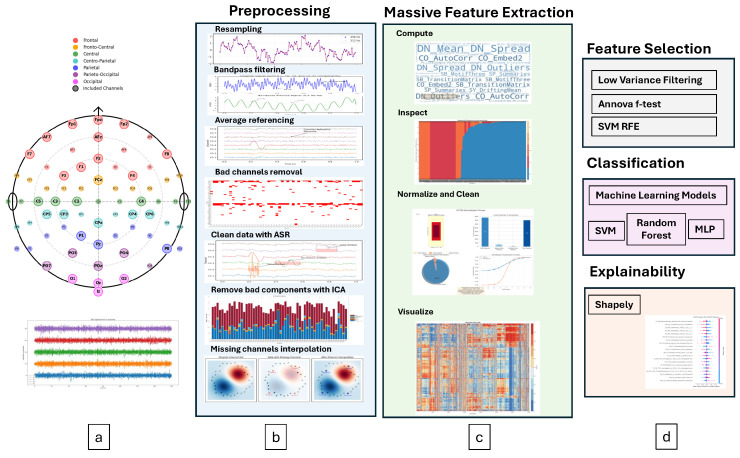
Proposed ASD prediction framework: (**a**) shows the electrode montage with 32 EEG channels used in this study, with color-coded brain regions. (**b**) shows the preprocessing pipeline to clean EEG signals. (**c**) Massive feature extraction after noise and artefact removal. (**d**) shows the feature selection, classification, and explainability components of the framework.

Our model not only achieves high accuracy but also provides insights into the most influential channels and features contributing to our model’s decision to detect ASD. The proposed framework is summarised below and shown in Figure 1:

1. Comprehensive feature extraction: We leveraged the highly comparative time-series analysis (HCTSA) engine (first study in this domain to the best of our knowledge) to extract a vast number of EEG-based time-series features spanning more than 10 domains, providing a highly detailed representation of neural activity.
2. Hybrid feature selection: Our method integrates both statistical filtering and machine-learning-based selection, optimising the most relevant features for classification.
3. High classification accuracy: By selecting the top 50 features, our pipeline achieves 100% 5-fold cross-validated classification accuracy, demonstrating the effectiveness of the proposed framework on this dataset.
4. Explainability via SHAP analysis: Unlike many black-box models, our approach provides interpretability using Shapley values, allowing identification of the most relevant EEG channels and features, enhancing clinical relevance and acceptability.

## 2. Materials and Methods

### 2.1. Dataset

A publicly available perfectly balanced dataset of 56 subjects (28 ASD, 28 typically developing (TD) controls) with an age range of 18.08–68.33 years (mean age = 504.21 months, SD = 147.44) and a mean Social Responsiveness Scale (SRS) score of 56.86 (SD = 13.49) [23] was used. The ASD group comprised 12 males and 16 females, while the TD group comprised 15 males and 13 females. Cognitive assessment was performed using the Matrix Reasoning subtest [24], and autism symptoms were quantified using the SRS [25]. EEG data was collected for a 150-s (2.5 min) period of eyes-closed resting state using a 64-channel Biosemi ActiveTwo system (Amsterdam, The Netherlands) with a sampling rate of 2048 Hz, impedance ≤ 25 kΩ, except for 9 participants for whom a 128-channel system was used. Before analysis, channels from 128-sensor montages were mapped according to the Biosemi 128-64 mapping file, such that the sensors were located in the same or similar locations to the 64-sensor montage and had the same channel names. This data is available from Sheffield University data repository (https://doi.org/10.15131/shef.data.16840351).

### 2.2. Preprocessing

Each participant has a different number of channels in the final dataset. For our framework, it is imperative to have equal and the same channels for all participants.

Preprocessing was carried out by EEGLAB in MATLAB (R2023b) [26]. The dataset was downsampled to 256 Hz from 512 Hz, due to the high computational demands of our feature-extraction engine. The data is then bandpass filtered from 0.5 to 30 Hz, for dominant frequencies in the resting-state EEG while minimising the computational burden [27]. Whilst gamma band activity (>30 Hz) has been implicated in ASD research [28], we focused on the 0.5–30 Hz range where the most robust resting-state biomarkers have been documented [9]. Future work may explore the inclusion of higher frequency bands. Average referencing is used to remove the reference electrode bias. Bad channels are then removed from the data using the EEGLab rejchan function that uses kurtosis to detect statistically abnormal channels. To remove artefacts (eye blink, muscle movements, line noise, heart noise), artifact subspace reconstruction (ASR) and independent components analysis (ICA) were applied sequentially. ASR was applied first to remove high-amplitude artefacts, followed by ICA for the removal of stereotypical artefact patterns [29].

Instead of interpolating all bad channels, we selected 32 channels that were present in at least 90% of participants after bad-channel removal. This approach ensures data quality whilst maintaining consistency across the cohort. For the few remaining participants missing one or more of these 32 channels, interpolation was performed using the spherical spline method [30]. This ensures that interpolation is minimum and only applied when necessary to standardise the channel set. The final dataset for feature extraction has the following channels based on the 10–20 internaiton system (as shown in the Figure 1 montage): Fz, C5, F7, O1, P8, AFz, CP4, FP2, FPz, O2, Oz, P1, POz, Pz, CP3, F3, PO3, PO4, C3, CP5, CP6, FCz, Iz, PO7, AF7, C1, CPz, F8, FP1, C4, F1, F4. This montage provides comprehensive coverage of frontal, central, parietal and occipital regions consistent with previous ASD neuroimaging studies [31].

### 2.3. Massive Feature Extraction

The HCTSA MATLAB package was used to extract features comprising over 7700 time-series features from each channel [32]. HCTSA is a comprehensive framework for time-series analysis that systematically compares thousands of features from diverse domains. Some of the salient feature domains include the time domain (mean, median, variance, skewness, kurtosis, autocorrelation, hurst exponent, Shannon entropy), frequency domain (spectral power, peak frequency, spectral entropy and flatness, wavelet energy and variance), nonlinear and entropy-based domain (sample/approximate/permutation entropy, Fisher information, correlation dimension), time–frequency-based domain (short time Fourier transform energy, wavelet coherence), and model-based domain (AR model coefficients, state-space model parameters). The feature space was reduced from 7755 to 7048 per channel before normalisation, after features with errors, NaNs, or infinite values, were filtered and cleaned following preliminary computation and visual inspection. In order to guarantee numerical stability in the ensuing feature-selection and classification phases, this cleaning step is crucial [32].

### 2.4. Feature Selection

Following the extraction of features from each of the 56 subjects’ 32 channels, the features were concatenated to produce a massive initial set of 225,536 features per subject (225,536 × 56 instances). A hybrid feature-selection approach was employed due to the enormous size [33]. Since low-variance features are unlikely to be helpful in differentiating between classes, they were first eliminated using a threshold of 0.001. The ANNOVA f-test method, which statistically measures variance between groups relative to variance within groups and selects features that best separate class labels, was used to further reduce the feature set to 10,000 features. It works well with a big feature set and is quick and computationally efficient [34]. Finally, recursive feature elimination (RFE) based on a linear support vector machine (SVM) was utilised, which recursively eliminates the least important features based on the selected classifier until a desired number of features remains. A step size of 100 was used to balance computational efficiency with selection granularity [35].

### 2.5. Machine-Learning Models

Three machine-learning models were used to classify ASD and TD subjects using a stratified 5-fold cross-validation process [36]. Stratified sampling ensures that each fold maintains the same class proportion (50%/50% ASD/TD) as the full dataset, preventing class-imbalance artefacts. In each fold, 80% of data (45 participants) was used for training and 20% for testing.

These models were selected based on their documented performance in EEG-based classification tasks and their complementary properties [37]. SVM is a supervised learning model that finds the optimal hyperplane to separate the available classes. A radial basis function (RBF) kernel was selected as it captured complex decision boundaries in high-dimensional spaces [38]. SVM has demonstrated a strong performance in neurophysiological signal classification [36]. The second model was random forest (RF), which is an ensemble learning method that constructs multiple decision trees and takes a majority vote for predictions [39]. A multi-layer perceptron (MLP) model was also included in our analysis, which is a neural network consisting of an input layer, hidden layers, and an output layer. It uses back propagation and gradient descent to optimise its weights and can learn complex patterns when trained properly [40].

Whilst deep-learning models such as convolutional neural networks (CNNs) and long short-term memory (LSTM) networks have shown promise in EEG analysis [41], and regularisation techniques (dropout, L1/L2 regularisation, data augmentation) can help mitigate overfitting, they typically require substantially larger datasets to learn robust representations. Given our sample size of 56 subjects, we opted for models with fewer parameters that were more appropriate for small- to medium-sized datasets [42]. Additionally, the selected models offer better interpretability through SHAP analysis, which is crucial for clinical acceptance [43].

Model hyperparameters were set based on common best practices: SVM used an RBF kernel with C = 1.0 and gamma = ‘scale’; random forest used 100 estimators with default parameters; MLP used two hidden layers (100, 50 neurons) with ReLU activation and an Adam optimiser. No additional hyperparameter tuning or bootstrapping was performed to avoid inflating performance estimates on this small dataset. Performance metrics (precision, recall, F1-score) were averaged across the five folds, with each participant appearing in the test set exactly once.

### 2.6. Explainability

There is a huge barrier to using machine-learning models in clinical settings due to their black-box nature. Explainability of those models may help reduce those barriers by understanding and interpreting those models, and fostering trust and accountability. Shapley Additive Explanations (SHAP) is such a technique that assigns an importance weight, based on the Shapley value, to each feature of a trained model for a particular prediction [43]. Shapley values, a concept originally from the game theory, is a method for fairly distributing the total gains or costs among players in a coalition [44]. In machine learning, players are the features and model prediction is the game, where the value is distributed among features. SHAP values have been successfully applied in medical diagnosis to identify the most important features driving predictions [45].

## 3. Results

The aim of this work was not only to develop a highly accurate ASD-prediction framework but also to identify prediction accuracy on different numbers of selected features from thousands of features belonging to different domains. For example, for an on-chip wearable device, the aim is to identify features and models that require the least amount of hardware resources. Although the feature-extraction process was computationally intensive, it was valuable to get insights into important features and channels for ASD detection for a real-time resource-sensitive application. We have tested our three machine-learning models (SVM, MLP, and random forest) using five-fold cross-validation on different numbers of features (1, 3, 6, 9, 15, 20, 50, 100).

The performance metrics (precision, recall and F1 score) of each model with different numbers of selected features are shown in Table 1. The performance of every model improved as the number of features increased. SVM produced a precision of 0.9 but a poor recall (0.43) with only one feature, suggesting a high number of false negatives.

Notably, all three models exhibited a marked decline in precision when using only three selected features compared with a lone feature, while recall improved. The tradeoff between false-positive and false-negative rates at various feature dimensionalities is reflected in this seeming paradox. Models that only use one feature are typically conservative, producing high precision but low recall (many false positives). Models become more aggressive in positive classification as the number of features rises to three, increasing recall at the expense of precision (more false positives). This illustrates how three features offer insufficient details for a certain classification, leading to erratic decision boundaries. With ≥9 features, performance stabilises and improves across all metrics, suggesting that robust ASD detection requires a richer feature representation.

For ≥20 features, all models demonstrated significant improvements, with SVM and MLP exhibiting precisions of ≥0.97. Notably, SVM and MLP both achieved perfect classification (precision, recall, and F1 score of 1.0) with ≥50 features, while random forest achieved 100% accuracy with 100 features. On the majority of feature-selection thresholds (1–20 features), MLP consistently outperformed both SVM and RF for balanced metrics like F1 score. The inherent capacity of MLP to represent intricate nonlinear relationships between features through its hidden layers may be the cause of this better performance. MLP demonstrated its efficacy with a comparatively small feature set by achieving an F1 score of 0.91 with nine features, as opposed to 0.84 and 0.78 for SVM and RF, respectively.

**Figure 2:**
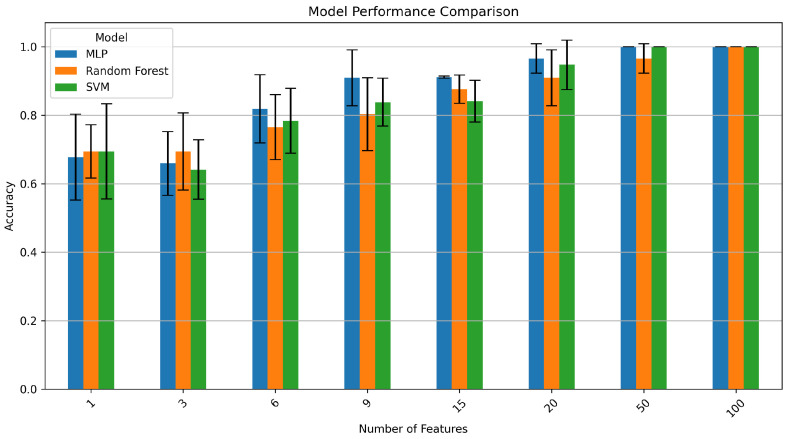
Model performance comparison across different feature selection thresholds.

Figure 2 displays the average model performance (accuracy) as a function of a given number of features. Increasing the number of features from one to twenty yields the biggest improvements in validation accuracy. The performance differences between the models, however, decreased at higher feature counts (50, 100), with all three achieving near-perfect or perfect classification. This convergence implies two things: first, the choice of algorithm becomes less important when the feature set is rich; second, when trained with an optimal feature set, simpler models like SVM could be used in real-world applications without compromising performance.

The 100% accuracy achieved with ≥50 features, while impressive, must be interpreted with caution given our sample size of 56 participants. This performance was obtained using a stratified five-fold cross-validation, which provides some protection against overfitting by ensuring each data point serves as a test sample exactly once. However, cross-validation on small datasets can still yield optimistically biased estimates of generalisation performance. The consistency across precision, recall, and F1 score suggests genuine discriminative patterns rather than pure overfitting, but independent validation on larger external cohorts is essential to confirm true generalisation capability.

**Figure 3:**
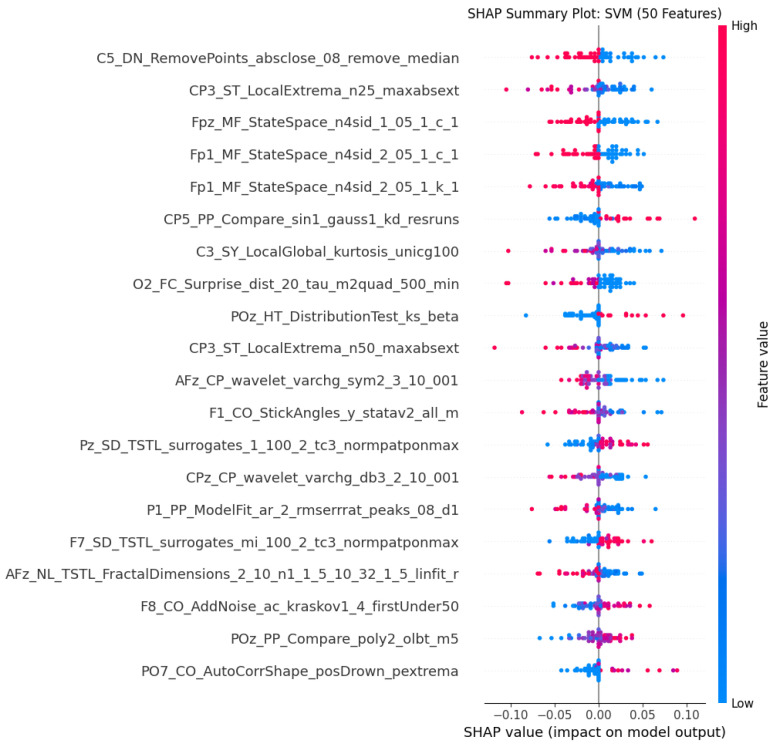
SHAP summary plot showing the top 20 channel–feature combinations contributing to ASD classification.

The SHAP summary plot in Figure 3 shows the top 20 channel–feature combinations that contributed the most when the SVM model was used with 50 features and obtained 100% accuracy. Each row in the plot indicates the specific channel–feature combination, and the position on the x-axis indicates the contribution to the model output (Shapley value). The Shapley value is an indication of how much each feature is contributing to increasing or decreasing the model output, where an increase is represented by (1-ASD) and a decrease is represented by (0-TD). The SHAP plot indicates that there is a wide distribution of channels, with diverse representations across brain regions. The most dominant channel representations are, however, the frontal and central regions, which is in agreement with the existing literature on abnormalities in the frontal lobe in ASD patients [18]. The most impactful features also come from diverse domains, such as the state-space modelling domain, where there is an indication of differences in the dynamical system properties in ASD; statistical features such as local extrema and local global; wavelet features; correlation; and distribution tests. The positive and negative values show that some features contribute to ASD classification when their values are high (red dots on the right), while others contribute when their values are low (blue dots on the right). This bidirectional effect demonstrates the complex nature of ASD-related EEG patterns. Moreover, the short range (−0.1 to 0.1) signifies that the model relies on many small contributions of features rather than a few strong ones, suggesting a distributed pattern of neural differences in ASD rather than a single dominant biomarker.

## 4. Discussion

Our study demonstrates that a massive-feature-extraction approach combined with hybrid feature selection can achieve high classification accuracies for ASD detection using resting-state EEG. The 100% accuracy obtained with 50 or more features using SVM and MLP models is encouraging but must be interpreted cautiously within the context of the specific dataset and highlights the need for external validation on independent, larger cohorts [46]. Previous EEG-based ASD studies with similar or slightly larger sample sizes have reported lower accuracies. For instance, Rogala et al. [14] achieved 86% accuracy on 36 subjects, whilst Tang et al. [13] reported 85.12% on a larger dataset. The higher performance in our study may be attributed to the comprehensive feature-extraction approach (>7000 features per channel) compared with more limited feature sets in previous work.

Our results compare favourably with recent studies in the field, though direct comparison is challenging due to differences in datasets, preprocessing, and validation strategies. Lalawat et al. [12] achieved 97.35% accuracy using deep learning on a 16-subject dataset. Whilst their accuracy is high, the extremely small sample size limits generalisability. Importantly, our approach achieves this performance while maintaining complete independence from target domain data during training; a critical advantage for practical deployment where target participant data may not be available beforehand, as highlighted in recent transfer learning work on EEG-based applications [47].

Most studies, for example [18,19], extract features from one or two domains. Our comprehensive feature-extraction approach captures information from multiple domains (time, frequency, nonlinear dynamics, and model based), which may explain the improved performance compared with studies focusing on single domain features. By extracting over 7000 features per channel and allowing the hybrid selection algorithm to identify the most discriminative subset, we avoid the potential bias of pre-selecting specific feature types. This data-driven approach may reveal unexpected biomarkers that would be missed by hypothesis-driven feature engineering [32].

In our work, we used a hybrid method for feature selection that included variance filtering, ANOVA F-test, and SVM-RFE. The advantage of a multi-stage feature selection method over a single-stage method is presented in [33]. The other feature-selection techniques that can be used for our purpose are filter methods (mutual information, chi-square test), wrapper methods (forward selection, backward elimination), and embedded methods (LASSO, elastic net) [34]. The selection of a multi-stage method is based on its computational efficiency and its proven effectiveness in high-dimensional data, especially in biomedical applications [35]. However, other methods may also be considered in future studies to see if they produce a better optimal set of features or better classification performance.

We selected SVM, random forest, and MLP as our classification algorithms based on their documented success in EEG-based classification tasks [36,41]. These models strike a balance between complexity and interpretability, which is crucial for clinical applications. Deep-learning methods, particularly convolutional neural networks (CNNs) and recurrent architectures like LSTMs, have shown impressive performance in EEG analysis [48]. However, such models typically require substantially larger training datasets to avoid overfitting. With 56 subjects, deep-learning approaches would likely overfit despite regularisation techniques [42].

The SHAP analysis revealed that the most discriminative features came from multiple brain regions and signal domains. First, the prominence of frontal and central channels aligns with the extensive literature documenting frontal lobe abnormalities in ASD, including altered frontal connectivity, executive function deficits, and social cognition impairments, providing neurobiological validation of our model [18]. Second, the diversity of discriminative feature types (state-space, wavelet, statistical) demonstrates that the ASD spectrum is due to multi-faceted alterations in neural dynamics rather than a single anomaly, which has implications in understanding ASD heterogeneity. In this context, for example, state-space model features, which characterise the dynamical properties of neural signals, suggest that ASD involves differences in the temporal dynamics of brain activity, consistent with theories of altered neural oscillations in ASD [31]. Wavelet features show time–frequency abnormalities related to atypical neural synchronisation patters and statistical features are connected with the altered temporal structure of brain activity. Third, the phenomenon of both high and low feature values contributing to ASD classification reflects the complex nature of neural alterations in ASD, which depicts that some individuals may show hyperactivity while others show hypoactivity in certain regions and frequency bands. Fourth, the distributed patterns of small contributions rather than a few dominant features suggest that ASD biomarkers may be better captured through multi-dimensional fingerprints rather than single markers, which could inform future biomarker discovery efforts.

Our study focused on univariate features extracted from individual channels. However, recent research has highlighted the importance of functional and effective connectivity in understanding ASD neurobiology [11]. Effective connectivity methods, which infer causal relationships between brain regions, provide even deeper insights into neural circuit dysfunction in ASD. Methods such as Granger causality, transfer entropy, and directed transfer function have revealed an altered information flow in ASD networks, particularly in frontal–parietal circuits involved in attention and executive function. Incorporating connectivity features alongside our univariate features could enhance model performance and provide richer neurobiological interpretation. This represents a promising direction for future work.

The potential influence of factors such as gender and age on ASD biomarkers is well documented in the literature [49]. While our sample size of 56 subjects (wide age range and relatively balanced gender distribution) precluded systematic stratified analysis by these demographic variables, future work with larger cohorts should investigate age and gender-specific biomarker patterns. This may also open up avenues for personalised diagnostic approaches.

### 4.1. Clinical Applications and Translation

The proposed framework has significant potential for future clinical applications. First, the framework may be used for screening patients in paediatric clinics and psychiatric clinics for ASD risk. This is particularly significant for resource-limited settings. Second, the SHAP method is easy to interpret, which may help build trust among practitioners. This is significant because trust is essential for the successful adoption of any framework or method. Third, the discriminative EEG features may be useful for designing low-cost portable devices for screening ASD. Fourth, the feature profiles may also have potential for treatment response or neurodevelopmental trajectories for patients already diagnosed with ASD. However, we would like to reiterate that significant validation work is required before the framework may be used for clinical applications. This work is an important proof of concept that has significant room for development before real-world application.

A major practical issue to consider is the extent to which our set of features and models would perform for a new set of completely independent participants. Whilst the cross-validation procedure does offer a limited measure of how well a set of features would perform in a more general case by ensuring each participant is a test case exactly once, the only way to truly measure the extent to which a set of features would perform for a new set of completely independent participants is to perform external validation. In the case of a larger set of participants, we would expect two possible cases to be true: (1) If the 50 features we have selected are indeed true biological markers for ASD and are generally consistent across the population, we would expect the high performance to translate to a new set of participants. However, we would still expect the accuracy to be less than 100%, simply due to the inherent heterogeneity of the disorder. (2) If the features we have selected are in fact dataset-specific artefacts and simply describe the specific characteristics of the cohort we have used in the present study, we would expect the performance to be significantly lower.

### 4.2. Limitations

Several limitations must be acknowledged. First, our sample size of 56 participants, while comparable to or larger than many previous EEG-based ASD studies, remains small for machine-learning applications and limits statistical power for subgroup analyses. Second, our dataset comes from a single source with specific acquisition protocols, which may limit generalisability to other EEG systems or recording conditions. Third, participants represent a specific demographic profile (mean age 42 years) and may not reflect the full autism spectrum, particularly children, who are the primary target for early diagnosis interventions. Fourth, we focused on resting-state EEG, which may not capture task-related or socially relevant neural alterations. Fifth, we limited our frequency range to 0.5–30 Hz, excluding gamma-band activity (>30 Hz), which has been implicated in ASD research but was excluded to reduce computational burden. Sixth, our perfect classification accuracy, while obtained through rigorous cross-validation, may partially reflect dataset-specific patterns and requires validation on independent cohorts. Seventh, we did not systematically investigate potential confounds such as medication history, comorbidities, or IQ, which could influence EEG patterns. Finally, the computational intensity of HCTSA feature extraction may limit real-time application without optimisation.

## 5. Conclusions

In this study, we introduced a novel framework for ASD detection using massive feature extraction from EEG signals coupled with a hybrid feature-selection method. Our presented findings demonstrate that an optimal subset of features can achieve 100% cross-validated accuracy on this dataset. However, we emphasise that these results, while encouraging, require careful interpretation within the context of our sample size (n = 56) and must be validated on larger, independent, and more diverse cohorts before clinical deployment. The high accuracy may partially reflect dataset-specific patterns, and we caution against over interpreting these preliminary results. Shapley analysis demonstrated that different brain regions and feature types were involved in the decision-making process. This also reinforces the value of a massive-feature-extraction approach. This multi-domain fingerprint may explain why our model achieves 100% accuracy with just 50 features, as it captures complementary aspects of brain-activity differences in ASD. Future research should pursue several directions to build upon these findings. First, validation on larger, independent datasets from multiple clinical sites and diverse populations (different ethnicities, age ranges, ASD severity levels) is essential to assess generalisation. Second, incorporation of functional and effective connectivity features alongside our univariate features may capture complementary information about neural circuit dysfunction. Third, development of streamlined feature sets optimised for portable, low-cost EEG devices could enable point-of-care screening. Seventh, comparison with regularised deep-learning models on expanded datasets would provide valuable methodological insights. Finally, an investigation of whether these EEG features can track treatment responses or predict intervention outcomes would enhance clinical utility.

## Figures and Tables

**Table 1:** Performance metrics of different models with varying number of selected features.

Model	Num of Features	Precision	Recall	F1 Score
SVM	1	0.90	0.43	0.57
	3	0.63	0.68	0.65
	6	0.82	0.78	0.78
	9	0.82	0.89	0.84
	15	0.90	0.81	0.83
	20	0.97	0.92	0.93
	50	1.00	1.00	1.00
	100	1.00	1.00	1.00
MLP	1	0.86	0.43	0.55
	3	0.64	0.72	0.67
	6	0.85	0.78	0.80
	9	0.90	0.93	0.91
	15	0.90	0.92	0.91
	20	0.97	0.96	0.96
	50	1.00	1.00	1.00
	100	1.00	1.00	1.00
Random Forest	1	0.75	0.56	0.64
	3	0.70	0.63	0.66
	6	0.86	0.68	0.74
	9	0.87	0.72	0.78
	15	0.88	0.88	0.86
	20	0.93	0.88	0.89
	50	0.97	0.96	0.96
	100	1.00	1.00	1.00

## Data Availability

The dataset used in this study is publicly available from the cited source.
